# UNDERSTANDING RACIAL BIAS AWARENESS ACROSS ADULTHOOD: THEMES OF BIAS, JUSTIFICATIONS, AND EMOTIONS

**DOI:** 10.1093/geroni/igae098.4319

**Published:** 2024-12-31

**Authors:** Jennifer Beatty, Patrick Hill, Jonathan E Doriscar, Seanna Leath

**Affiliations:** Washington University in St. Louis, St. Louis, Missouri, United States; Washington University in St. Louis, St. Louis, Missouri, United States; Northwestern University, Evanston, Illinois, United States; Washington University in St. Louis, St. Louis, Missouri, United States

## Abstract

This project employed a mixed-methods approach to analyze how age-diverse White Americans (N = 345; age range: 19 to 85) make meaning of specific instances of personal racial bias. Across three data collections, that targeted younger, middle-aged, and older adults, we asked participants to write about a time when they were racially biased. In the samples, 98% of younger adults, 91% of middle-aged adults, and 86% of older adults indicated specific instances of racial bias, and many indicated antiblack bias. Age-diverse adults frequently assumed that racial out-group members were criminal or deviant or assumed that those individuals had an undesirable culture. The participants also often indicated numerous justifications and emotions about their bias. For example, common justifications across all ages included perceived threat or the ambiguity of the situation. Specific emotions and justifications only differed moderately by age. Our findings indicate that while age-diverse White adults may make meaning of their bias in different ways, they tend to do so with self-conscious emotions and bias justifications that do not mitigate bias. Critically, middle-aged and older adults were less likely to indicate emotions at all in their reports. These findings may reflect a positivity bias among older adults who are less willing to highlight or remember their negative emotions or it may speak to the possibility that they may feel less fear in intergroup settings. The current work offers insights into more personalized approaches to bias regulation by integrating specific bias contexts with bias justifications and emotional awareness.

